# Lectures

**Published:** 1844-10-05

**Authors:** Benjamin Collins Brodie

**Affiliations:** Consulting-Surgeon of the Hospital


### CLINICAL LECTURES AND REPORTS.

LECTURES

*Delivered in the Theatre of St. George's Hospital, in the session* 1843–44,

BY SIR BENJAMIN COLLINS BRODIE,

Consulting-Surgeon of the Hospital.

NON-SCIRRHOUS TUMOURS OF THE BREAST.

*On the use of mercury in syphilis. Treatment of syphilis without mercury. Cases in which mercury should or should not be given.*

Gentlemen,—The observations which I addressed to you in my last lecture were made on the supposition that care would be taken to distinguish scirrhous and other malignant tumours of the breast from those of a non-malignant character. I consider it unnecessary to call your attention to the diagnosis of different tumours, but I am anxious to impress upon your minds that you must be careful to learn this for yourselves from other lectures. When a practitioner tells me that he has been particularly successful in the operation for scirrhous tumours of the breast, I am always satisfied that there has been a want of accuracy in the diagnosis. I remember a gentleman stating that he had performed this operation ten times, and that the disease had not returned in a single instance. No very experienced surgeon would have made that statement, but I subsequently saw a tumour which this gentleman was going to remove, and it was nothing more than a common chronic abscess of the breast which he had denominated scirrhus.

I shall now call your attention to another subject—the administration of mercury in cases of syphilis. I shall not enter into detail either as to the mode of its exhibition or the cases in which recourse should be had to it; but I propose to make some general observations, which, at this time, when so much difference of opinion prevails as to the use of mercury, and there is so much random practice in its employment, may be serviceable to you in the beginning of your profession.

Mercury was used in case of syphilis very soon after the disease was first recognised in Europe. I believe that from within three or four years after the siege of Naples, where it was supposed it first broke out, through good report and through evil report, in spite of the too strong prejudices of some in its favour, and of others against its use, mercury has maintained its general reputation in the profession up to this day. At different periods, however, other remedies have been proposed for the cure of venereal disease. The late Sir Wm. Fordyce wrote a pamphlet for the purpose of proving that it was to be cured by sarsaparilla. An army surgeon, Mr. Grant, wrote a pamphlet in favour of opium; another practitioner has cured it by ammonia, and others have spoken highly of nitro-muriatic acid. Many other remedies have been proposed as a substitute for mercury, which it is not necessary for me to enumerate. In hot climates—Spain Portugal, the West Indies, and the islands of the Pacific Ocean,—syphilis was said to be cured without the aid of a particle of this remedy. But in opposition to what I have just mentioned there was, in the beginning of this century, a prevailing notion that mercury was a specific for syphilis, and that it was never cured without it. The late Mr. Abernethy, in his work on what he terms pseudo-syphilis, lays it down as a rule that syphilis is uniformly progressive if mercury be not administered, and he said of every disease that came before him in which the symptoms improved without the aid of mercury, "this cannot be syphilis." He gave no reasons for this extraordinary assumption—it was a complete *petitio principii*—a begging of the question, and this illogical conclusion at which he had arrived, was sufficient to destroy the value of this part of his works. Not long after this opinion had been published by him and was maintained generally throughout the profession, a friend of mine, the late Mr. Rose, who subsequently became surgeon to this hospital, instituted a series of experiments on the subject of the treatment of this disease. He had ample opportunities of carrying these on; for he was surgeon to one of the regiments of Guards, and soldiers associating with the lower order of prostitutes, I need hardly say are very liable to become affected with syphilis. For one or two years he treated every soldier that came into the regimental hospital, suffering under any form of syphilis, without mercury. I saw these cases, and every now and then watched their progress with him. Every sore upon the organs of generation was cured under his management without the employment of this agent. It is true that many of these sores were not venereal, but many of them were of that character; and the hardness which was left behind disappeared without resort to mercury. Many of these patients never had secondary symptoms, which may be attributed to the sores not having been venereal; but in some cases, where secondary symptoms appeared, they were slight, and others severe, exhibiting nearly the usual character; but whatever they were they yielded without this agent. In two or three cases where there was inflammation of the iris, and mercury was necessary in order to save the eye, he employed it. Mr. Rose, therefore, came to the conclusion, which these cases seemed to justify, that the disease was one which would get well even if mercury was not used. Other army surgeons repeated these experiments, and arrived at the same result, and I believe that the disease is now treated in the army to a considerable extent in this manner.

Now, these observatinos led a certain part of our profession to a view of the subject entirely different from that which they before entertained; and some began to contend that mercury did a great deal of harm, and was in itself a worse disease than the one it was intended to cure. With respect, however, to recovery from syphilis without the aid of mercury, I do not believe that you can apply a rule, drawn from the observation, of what occurs in soldiers to society at large. We find that the effects of disease in all cases depends very much on the kind of constitution that has to sustain it. Students from the country, on coming here, have frequently expressed their astonishment at the difference in recovery from compound fracture in the hospital and in those places in the country where they have seen it. But here the occurrence takes place in one kind of constitution, and there in another. When the cholera visited London it destroyed 300 out of more than 1,500,000 inhabitants; in Sunderland it carried off a large proportion of the population; and in Paris I think the mortality was about one in thirty. Here the cholera did not destroy the affluent classes, but those who were ill-fed, ill-clothed, and were breathing a poisonous atmosphere, and they sank under it with great rapidity. So I apprehend it to be with syphilis. Soldiers are men with strong constitutions, and are in good health, otherwise they would not be received in the army. They are not much advanced in life; they are sent to the regimental hospital, and are there kept constantly under the eye of the surgeon, dieted as he pleases, and their general health is attended to in every respect. They are not allowed to be exposed to atmospheric changes of the weather, and, in short, from their constitution and the situation in which they are placed, they may be supposed to have the power of throwing off morbid poisons which would not be thrown off by others. Experience and practice will, I think, fully confirm these observations. I know that in this hospital I have tried to treat syphilitic patients without mercury with very little success indeed, and that in private practice the attempt would prove altogether a failure. Sir Wm. Wimpress, who was surgeon-major to the Coldstream Guards, but has now retired from service, saw a great deal of syphilitic practice, and he told me that he could manage the cases of privates in this manner, but not of officers. When Mr. Rose entered into private practice he thought that he could apply the same rule there which he had carried out among the soldiers, but he found that he could not, and he was compelled, like other surgeons, to give mercury. In cases where he endeavoured to avoid its exhibition he found that he was continually beset with difficulties.

With regard to the other point I mentioned,—the opinion that mercury very often tends to aggravate the disease instead of doing good,—I know that its injudicious use will do harm, but I am satisfied that that is not the result when it is wisely administered. It has been said that there is no disease of the bones where mercury is not given. I know that in patients who are treated by mercury there is a greater chance of disease of the bones than there was in Mr. Rose's patients, to whom it was not exhibited; and I know that when given for liver complaints and for diseased testicle it may produce nodes. But, admitting this to be true, I am quite sure that syphilis will run on till it produces nodes, by which I mean disease of the bones, even where no mercury has ever been given. I will state a case in point. A gentleman had a chancre which no one doubted to be venereal; he took no mercury and it healed up. I do not remember exactly what symptoms followed, but when I saw him, in consultation with Mr. Rose, a couple of years afterwards, he had extensive disease of the bones of the nose, which was still advancing; we agreed that the best thing we could do was to put him under the influence of mercury, of which he had never taken a grain, and try whether or not it would stop the disease. He was to be furnished with lodgings in London, for the purpose of going through a mercurial course, but he had a fit of epilepsy, and then another, and that was followed by a third, after which he became maniacal and died. I do not know that there was any post mortem examination, but neither Mr. Rose nor myself doubted that the disease had crept up the ethmoid cells, attacked the ethmoid bone, and affected the brain and its membranes. I saw another case treated without mercury. A patient had a primary sore, of which he got well, but a few months afterwards there was pain of the limbs, which were considered neuralgic, and by and by there was a node on the shin and another on the elbow. He had never had any disease prior to the chancre, and we could not but suppose that the virus had entered the system, and the secondary symptoms being passed over, had gone at once to the bones. The conclusion of the case was very remarkable; the patient got entirely well with sarsaparilla, no mercury being given.

I am sure that experience proves to me, and it will to you, that we find no remedy having the same power to extinguish venereal disease as mercury, but then it must be not only judiciously administered at the time, but care must be taken that it is only employed in proper cases; it may do great harm if it be improperly used. There is nothing remarkable in this fact. With the exception of sarsaparilla, I do not know of any medicine that will do great good that may not act as a poison. I saw a gentleman very nearly killed by an over-dose of quinine; the same circumstance has occurred from iodide of potassium, and many persons have been destroyed by arsenic. You are not to suppose that you are to administer mercury at random in all cases, but the general rule is that in cases of syphilis it is to be exhibited, and I shall endeavor to point out briefly, not the cases in which you may give it, but the exceptions to the rule.

First of all, there are persons of a certain delicate constitution, of a scrofulous disposition, and who are disposed to phthisis. You would not give mercury to a man of this kind until you were quite sure that it was absolutely essential; nevertheless, there are persons of a scrofulous tendency who are best treated by this means. If mercury be an evil, syphilis is still a greater one. In scrofulous persons local diseases are especially developed after the system has been effected by a morbid poison. If they are disposed to phthisis they will have tubercles in the lungs after scarlatina, measles, and small-pox; and it is just the same after syphilis. You find enlargement of the glands of the neck take place whenever the system is disturbed by syphilitic virus. and here mercury is not to be exhibited unless you are sure that it is wanted. But if there be syphilis it is better to give it than to let the disease have its course; it must, however, be administered with great caution, in moderate doses, and the patient must be carefully watched all the time.

Persons who appear to be in strong and vigorous health are not always good subjects for mercury. Many persons of this description have been living irregularly, drinking a great quantity of wine, and mercury is very likely to disagree with them and produce great mischief. True it is that the poison of syphilis will do the same; it will often produce frightful symptoms and the most intractable diseases; but it is better to put off the use of mercury for some time until you can improve the constitution. If mercury be exhibited under such circumstances you have two evils to encounter, but by withholding it you have only one. If you wait, put the patient on a better system of diet, make him live a more regular life, and attend to the generul health in all respects; you may then administer mercury with advantage, and probably cure the case.

There are some persons in whom, for reasons we cannot explain, mercury always acts as a poison. They certainly are few in number, but you cannot tell beforehand who they are, and therefore every person should be carefully watched to whom you administer mercury. Where there is a great deal of inflammation in the neighbourhood of a primary sore it is scarcely ever right to have recourse to mercury in the first instance; for the probability is that it will produce sloughing. You must combat the disease by bleeding, purging, and other means; and it is better to patch up the sore as well as you can, and let the disease go on until it has produced secondary symptoms, than to give mercury to a patient under these circumstances. In cases of phagedenic and sloughing chancre, where the condition of the chancre depends on the patient's constitution, mercury, if given in the first instance, will aggravate the disease, and make it spread more rapidly than it would otherwise do.

But there are cases in which the phagedena depends on the intense action of the venereal poison, as I shall hereafter explain, and in that case mercury may be exhibited.

You will sometimes find that in the case of secondary symptoms, mercury, instead of acting upon them and curing them, disturbs the general health; the symptoms increase, and the more you give the worse they become. This arises from the patient being in a bad state of constitution, which state of the constitution may depend on causes neither under your control nor that of the patient, but on the patient having taken mercury in an injudicious manner. Under these circumstances you must not continue this agent, but leave it off, and he may then recover; nevertheless you may require to revert to it at last. In order to illustrate this observation, I will mention a case. A man was brought into this hospital with sore throat, and a phagedenic eruption, having the character of syphilitic eruption, in different parts of the body, in a state of painful ulceration. He looked exceedingly ill, and I found that he had been taking mercury in large quantities, under a private practitioner, for five months. His gums were extremely sore when he came here, and the more mercury was pushed the worse he became; I therefore left it off, and gave sarsaparilla, and in a few months the eruption disappearing, he left the hospital. But after the lapse of a few months he came in again with sore throat and ulceration, having taken no mercury in the interval. I gave sarsaparilla a second time, and with the same beneficial effects, but the eruption did not disappear so rapidly as in the first instance. In the course of three or four months he again came in, and the ulceration was again spreading, accompanied with sore throat. I resorted to sarsaparilla a third time, and the symptoms went away, but more slowly than on either of the previous occasions. Towards the conclusion of the time that he was in the hospital, he laboured under inflammation of the iris, for which I gave him oxymuriate of mercury, and he got well. Three or four months after this the disease again broke out, the ulceration re-appeared and spread, and the sore-throat returned. He now went into the Lock Hospital, under the care of Mr. Blair. This was fourteen months after he first came to St. George's; he had taken no mercury during that time, except for the iritis, and Mr. Blair now very properly put him under a course of mercurial inunction, and I believe he was permanently cured. If I had done this when he first came here I should probably have killed him. I might mention a great many other cases to illustrate these observations.

Now, I have said that in the great majority of cases mercury is the best remedy you can employ for the cure of syphilis, but then care must be taken that it is properly and judiciously administered. There are different ways of exhibiting mercury; it may be given internally by pills; it may be used in the form of ointment, or by fumigation. The mercurial preparations that may be given internally are various—blue-pill, mercury with chalk, calomel combined with opium, Plummer's pill, iodide of mercury, bichloride of mercury, and some other forms.

I have often given mercury internally in the shape of pills. When you want to affect the system rapidly, as in iritis, pills are preferable, because the mercury affects the system sooner. A patient labouring under iritis is in danger of going blind, and you must remove it as soon as you can. You effect this much sooner by giving calomel and opium than by using mercurial inunction, and in slight cases the disease may be cured by mercury administered internally. There are a good many patients so circumstanced that they cannot take it in any other manner; at other times you are indifferent about the mode of administration; and in some cases you are compelled to give it internally against your inclination. Thus, upon the whole, there are a good many cases in which mercury will be exhibited internally.

But if you inquire which is the best way of giving mercury in cases of syphilis where the symptoms are not of the very mildest character, I must say that mercurial inunction is infinitely to be preferred to mercury taken by the mouth. Mercurial inunction, however, is dirty, laborious, and troublesome, and it makes the case public to the family in which the man lives. For these reasons it will be objectionable to the patient; but it has this advantage, it is much less liable to gripe and purge, and it cures the disease a great deal better. It does not damage the constitution half so much as mercury taken by the mouth; nay, I will go so far as to say that, except in the very slightest cases, you really cannot depend upon any mercurial treatment effecting a certain cure, or even giving a good chance of it, by any other means than inunction. You may very often patch up the disease by giving mercury internally, but it will return again and again, and you may cure it at last by a good course of mercurial ointment. But especial care must be taken that this is properly applied. If it be left to a patient he will rub it in for five minutes or so, whereas it requires to be rubbed in before the fire for three-quarters of an hour ere it enters; but by and by the friction may be continued for a shorter period. Where the symptoms are of the mildest character it is desirable that the patient should, if possible, be confined to the house. Mr. Pearson observed, long since, that going into the fresh air would undo the effect of mercury, and I never will be responsible for thoroughly eradicating the disease where the patient is at all exposed to cold, and where he does not lead a most careful and regular life.

In all cases where you employ mercury, you have two objects in view—first, to cure the present symptoms, and, secondly, to prevent their return. It appears to me that at the present day a great number of practitioners keep the first object only in view, and lose sight of the second. I have repeatedly seen persons who have taken mercury for chancre; it has healed in a fortnight, but a hard base has been left, and then in nine cases out of ten there has been secondary symptoms. If it be taken for a primary sore the patient should never leave it off until the hard cicatrix has disappeared. You must exhibit it until the sore has healed, and for some time afterwards; and the same plan must be pursued with reference to the secondary symptoms, or they will return. When the eruption has disappeared from the body it must be used as a prophylactic to prevent the return of the disease, for probably another month.

I should say that if a patient be confined to the house, or only allowed to go out a little once or twice a day, and if he be made to rub in mercury, and continues it for some time after the symptoms have subsided, the case being carefully watched, you will, in most instances, make a real and permanent cure of the disease. This is not the way in which it is administered by many practitioners now, but it is the mode in which it was done formerly. You must not suppose that we have made an advance in all departments of surgery; on the contrary, I am sure that in some we have gone back. I am satisfied that the mercurial treatment of syphilis, as employed by the late Mr. Pearson during a great part of his life, was as nearly perfect as possible, and it was much more successful than the less careful treatment of modern practitioners. Mr. Pearson was surgeon to the Lock Hospital, and having no general hospital to which to attend, the powers of his mind were very much devoted to this disease and to its treatment; and the practice which I have now recommended was that which he adopted. I had an opportunity of meeting him a great deal when I was first entering into practice, and I am satisfied that his mode of treatment was eminently successful. In his work on "Materia Medica" there is an article on syphilis, in which there are many excellent observations on mercury, treating the subject in detail in a way in which it is not my intention to do at present; but I refer you to that article as being well worthy of perusal.

Wherever you can, in the treatment of syphilis, make the patient take mercury in the form of unction if possible. It is the best plan to pursue in all cases, although it is not necessary in all cases; but where the symptoms are severe, and a long course is required, it is the safest mode of proceeding.

I will avail myself of this opportunity of stating the class of cases in which you may employ mercurial inunction with the greatest advantage. Children, when born, sometimes labour under syphilis, the father or mother having been affected with it—perhaps the father and not the mother. The child at birth looks thin, and is of small size, and instead of thriving it becomes still thinner. At the end of three weeks it is covered by a nasty scaly eruption; there is a sort of aphthæ in the mouth, and chaps about the lips and anus. I have tried different ways of treating such cases. I have given the child grey powder internally, and given mercury to the wet-nurse. But mercury exhibited to a child by the mouth generally gripes and purges, seldom doing any good; and given to the wet-nurse it does not answer very well, and certainly is a very cruel practice. The mode in which I have treated such cases for some years past, has been this: I have spread mercurial ointment, made in the proportion of a drachm to an ounce, over a flannel roller, and bound it round the child once a day. The child kicks about, and the cuticle being thin, the mercury is absorbed. It does not either gripe or purge, nor does it make the gums sore, but it cures the disease. I have adopted this practice in a great many cases with the most signal success. Very few children recover in whom mercury is given internally, but I have not seen a case where this method of treatment has failed.

Mercurial inunction may be used in certain cases in which were mercury taken internally it would do absolute harm. For example, a gentleman had a nasty phagedenic sore upon the penis; it could not be said that he was in ill health before, and therefore there was some reason to believe that the disease was spreading from the intensity of the venereal poison. He had taken calomel and opium until the gums were sore, and he was decidedly worse under it. The disease destroyed a great part of the glands, and evinced no disposition whatever to stop. It resisted all modes of treatment until he was put on a course of mercurial inunction; its progress was then arrested directly, and the sore healed with great rapidity. I have seen several instances of the same character.

Another mode of administering mercury is by fumigation, and this may be applied either locally to a part, or generally to the whole body. The patient is to sit in an apparatus like that used for sulphur baths, but instead of sulphur being thrown on a hot iron, black oxide of mercury is to be used. The patient may be affected very speedily by allowing him to hold his head inside the bath for two or three minutes, so that he may imbibe the mercurial vapour. I have employed this with success in several cases where it was my object to affect the system as quickly as possible, but I have found that Mr. Pearson's objection to it is well founded, namely, that it is difficult to regulate the action of the mercury. You may affect the system too much or too little, and you may be taken by surprise by the patient's gums becoming all at once excessively sore. With reference to the effect of mercury on the system generally, I believe it is always better that the gums should be made a little sore, and that there should be some degree of salivation. You cannot depend upon it when employed in syphilis, unless these effects are produced.

But, as I have already said, there may be cases in which mercury may not be proper at all, and in which there are reasons for doing without it if you can. In some individuals in private practice, as well as among soldiers, the affection will be thrown off by the patient's own constitution. In a great many instances slight symptoms will disappear merely by the improvement of the general health. A gentleman had a well-marked venereal eruption. He was in London, and was about to take mercury. He was called to go into the country, and I ordered him to let the mercury alone for the present. He had not been in the country air long before all the symptoms left him. Cases like these are recorded in Mr. Abernethy's book, and they led him to say that they were not cases of syphilis. After a patient has passed through a mercurial course, it is not sufficient to tell him that his disease is at an end. It is very important that he should be kept in good health. If, after the disease appears to be eradicated, the health is broken down, the disease may return at a considerable distance of time. After a mercurial course it is well to put the patient through a course of sarsaparilla, to remove the debilitating effects of the mercury itself from the constitution. I will mention a case to show how much depends on the state of the general health. A gentleman had secondary symptoms, and I put him through a course of mercurial inunction for ten weeks. He was confined to the house, and most carefully attended to, and took mercury for some weeks after the eruption had disappeared. He seemed to be quite well, and went abroad and continued so; but at the end of a year, being in Lisbon, he went out, got his clothes wet, and took cold. This was followed by a severe attack of erysipelas, and a Portuguese doctor very indiscretly bled him to a large extent, and an enormous abscess formed. His health became completely broken down, and he had now a return of the venereal disease, the symptoms being worse than they were before. When his health had improved, a surgeon in Lisbon put him under another course of mercury, and cured him.

In cases where the symptoms are aggravated by the use of mercury, they may be removed by sarsaparilla; in other instances they will subside under the use of iodide of potassium. It is now very much the custom to administer the latter in cases of syphilis. No doubt it is an excellent remedy in some cases, and it comes in to your aid extremely well where you have reasons for not giving mercury; but if you ask me whether you can rely upon iodide of potassium as well as upon mercury, I say, No. You may remove slight symptoms by giving it for a time, and severe symptoms by exhibiting larger doses; but in the latter case, so far as I have seen, it does not make a permanent cure, for the symptoms return again. As a prophylactic it is not to be compared with mercury.

I have spoken of the necessity of administering mercury, not only till the symptoms are relieved, but for a considerable time afterwards. You may inquire whether a long course of mercury will not injure the constitution more than a short one. Undoubtedly it will, but that is the very reason why you should give a long course at first. I will explain myself. If you exhibit a short course the disease is sure to return; you administer a second course, and the disease returns again, and thus you have repeated courses. Not only is the system weakened by the disease, but whenever it returns it assumes a more formidable character. But if you put the patient through a long course in the first instance, the frequent recurrence to the use of mercury will be unnecessary. A patient who takes mercury for a month will probably never require it again; but if he takes it only for a fortnight he has secondary symptoms, and then he will require to take it for four weeks, so that that which is a short course at first is a long one in the end.

With these observations I conclude the course of lectures which I undertook to give during this winter, but before we part allow me to make a few remarks with respect to the lectures themselves. When I resigned my hospital appointment, some three or four years ago, I could not but recollect that it was here that I had been able to lay the foundation of what little knowledge I may have attained in the profession, and that some of the happiest hours of my life had been passed within the walls of the hospital, in friendly intercourse with the pupils, and that among them I had found some of my sincerest and kindest friends. I felt, at the time of my resignation, that I owed a debt of gratitude which it was my wish, as far as I could, to pay. Under this impression I offered to the weekly board and to the medical officers to deliver this annual course of lectures.

It has been my endeavour to give you some information, which, of however little value it may be to those experienced in surgery, may, I hope, be of use to you who are younger men. But I have had another object in view in the construction of these lectures. They have been entirely practical, and, with hardly any exceptions, have been drawn from my own observation and experience. I wished to set you an example of what your own mode of study ought to be. In these times there is a great quantity of medical literature, such as it is. There are books on specific diseases, dictionaries, cyclopædias, compendiums, and manuals, of all kinds; and nothing is more easy than for a person with a tolerable memory to look into books and learn by heart the prevalent doctrines and opinions of the day, and then to be able to discourse on those subjects as if he really understood something of them, and to go and pass what is called a good examination; that is, to answer every question that is put to him. You may be successful in qualifying yourselves in this manner, but depend upon it, it will be of no avail to you in future life. A man who gets up this sort of knowledge from books, is good for nothing.

He goes to the bed-side of a patient, but he knows nothing either of the disease or of its treatment, and he is, therefore, in doubt about it. He has not that confidence in himself which enables him to take every responsibility, and which medical practitioners must do in difficult cases. You must, in order to be qualified for the situations which you are hereafter to fill in life, gain your knowledge, not from books, but from your own investigations. I do not say that you are not to look into books and to read them, but it should only be done in conjunction with practice. If you have a particular case before you, refer to a good book, and that will enable you to examine it far better than you would otherwise do; but the principal thing is to observe for yourselves. This remark applies to anatomy, to surgery, and to physic. You may get up anatomy by being examined by your teachers, by learning books by heart, and appear a very good anatomist to the man who examines you; but that knowledge will be of no service whatever in practice. No anatomical knowledge is of any use excepting that which you obtain by seeing the parts in the lecture-room and then examining them for yourselves and by your own hands in the dissecting room. I can assure you there is no other anatomical knowledge worth having, and the man who has qualified himself merely to pass an anatomical examination in the way to which I have referred will find that he has no chance whatever when he comes into competition with one who has made himself an anatomist in the proper way. So it is with respect to hospital practice; you must look at cases and study them for yourselves. Examine the cases in the morning and refer to books in the evening, otherwise you will have no useful knowledge. Consider the observations which drop from the medical officers, compare what they say with the living person, and take notes with your own hands. No person can learn either medicine or surgery who does not take notes, for it is the only way to obtain that knowledge which is necessary in practice.

I take the liberty of making these observations, not that you particularly need them, but they may be of use to younger persons in the profession. The way which I have pointed out is the only one in which you will be enabled to succeed in your profession, and to practice it with comfort to yourselves and advantage to the public. In fact, I think that very few will get into practice at all who do not pursue the study of the profession in the practical manner which I have suggested. I offer these remarks with an entire feeling of friendship, and with the most earnest wish that you may be successful in your profession and do honour to this school.

The worthy baronet then retired amid the acclamations of the pupils.—*London Lancet.*

